# A randomized controlled trial to examine the impact of a multi-strain probiotic on self-reported indicators of depression, anxiety, mood, and associated biomarkers

**DOI:** 10.3389/fnut.2023.1219313

**Published:** 2023-08-31

**Authors:** Kylie E. Walden, Jessica M. Moon, Anthony M. Hagele, Leah E. Allen, Connor J. Gaige, Joesi M. Krieger, Ralf Jäger, Petey W. Mumford, Marco Pane, Chad M. Kerksick

**Affiliations:** ^1^Exercise and Performance Nutrition Laboratory, Department of Kinesiology, College of Science, Technology, and Health, Lindenwood University, Saint Charles, MO, United States; ^2^Increnovo LLC, Milwaukee, WI, United States; ^3^Probiotical srl, Novara, Italy

**Keywords:** probiotic, gut-brain, quality of life, mood, serotonin

## Abstract

**Objective:**

To examine the efficacy of supplementing with a multi-strain probiotic (MSP) on changes associated with mood, anxiety, and neurotransmitter levels.

**Method:**

In a randomized, double-blind, placebo-controlled fashion, 70 healthy men and women (31.0 ± 9.5 years, 173.0 ± 10.4 cm, 73.9 ± 13.8 kg, 24.6 ± 3.5 kg/m^2^) supplemented with a single capsule of MSP (a total daily dose of 4 × 10^9^ live cells comprised of a 1 × 10^9^ live cells dose from each of the following strains: *Limosilactobacillus fermentum* LF16, *Lacticaseibacillus rhamnosus* LR06, *Lactiplantibacillus plantarum* LP01, and *Bifidobacterium longum* 04, Probiotical S.p.A., Novara, Italy) or a maltodextrin placebo (PLA). After 0, 2, 4, and 6 weeks of supplementation and 3 weeks after ceasing supplementation, study participants completed the Beck Depression Inventory (BDI-II), State-Trait Anxiety Inventory (STAI), and Leiden Index of Depression Sensitivity (LEIDS-R) questionnaires and had plasma concentrations of cortisol, dopamine, serotonin, and C-reactive protein determined.

**Results:**

BDI, STAI, and total LEIDS-R scores were reduced from baseline (*p* < 0.05) with MSP supplementation after 4 and 6 weeks of supplementation and 3 weeks after supplementation while no changes (*p* > 0.05) were reported in PLA. When compared to PLA, MSP scores for state anxiety, trait anxiety, and LEIDS-R (hopeless, aggression, rumination, and total score) were significantly lower (*p* < 0.05) after supplementation. Plasma serotonin concentrations in MSP were increased from baseline after 6 weeks of supplementation and 3 weeks after ceasing supplementation. No changes (*p* > 0.05) in plasma dopamine, C-reactive protein, or cortisol concentrations were observed between groups.

**Conclusion:**

MSP supplementation resulted in widespread improvements in several questionnaires evaluating mood, anxiety, and depression in young, healthy men and women. MSP supplementation increased serotonin increased after 6 weeks of MSP supplementation with no change in dopamine, C-reactive protein, or cortisol.

**Clinical trial registration:**

https://classic.clinicaltrials.gov/ct2/show/NCT05343533, NCT05343533.

## Introduction

Recent scientific investigations have confirmed the presence of an established gut-brain axis, an integrated network of bidirectional communication that involves contributions from immune, neural, and endocrine systems (1–4). In this axis and as explained in a recent review by Ahmed et al. (5), bacterial communities found in the intestinal microbiota signal the brain through an associated network by modulating activation of sympathetic and parasympathetic intestinal neurons (6, 7), education of the immune system (8) and production of several neurotransmitters (9–11) and gut hormones (12, 13). Moreover, bacterial communities and their metabolites (e.g., short-chain fatty acids, etc.) can go on to function as neuromodulators (14, 15) while impacting neuroinflammation (16–18), inflammatory modulators (19), energetic regulation for the host (20, 21), and are implicated in brain functions associated with neurodevelopment (22) and maintenance of the integrity of the blood–brain barrier (5). Additionally, germ-free mouse models have illustrated that the gut microbiota is crucial for normal brain development and behavior (23, 24) into adolescence (25). For these reasons, strategies to influence or alter the intestinal microbial environment may be employed to favorably impact mood and cognitive function. Probiotics, defined as live microorganisms that, when administered in adequate amounts, confer a health benefit on the host (26), are a notable intervention for influencing the microbial environment. Initial probiotic research in animals suggested favorable outcomes whereby probiotic administration seemed to favorably impact different aspects of cognitive function (27), anxiety (6) and depression (6). Likewise, human investigations have provided additional evidence to support the potential for probiotics to favorably influence the function and communication of the gut-brain axis. For instance, a functional magnetic resonance imaging (fMRI) study demonstrated that 4 weeks of probiotic use in 39 healthy women impacts regions of the brain involving emotional processing (28). Another four-week investigation using a multi-species probiotic demonstrated a positive effect on cognitive reactivity to sad moods in 40 healthy adults (29). Marotta et al. (30) expanded upon these findings when they reported that supplementation with a multi-strain probiotic mixture for 6 weeks in 38 non-clinical healthy volunteers favorably impacted mood by reducing depression and anger, while improving fatigue and sleep quality, and little change on personality attributes.

As evidence accumulates demonstrating the potential positive impact of probiotic use on mood, depression, and anxiety, investigations have attempted to link possible mechanisms to the functional outcomes. While human research is limited, a growing interest is evident to explore outcomes associated with stress modulation, neurotransmission, and inflammation and how these changes regulate and influence the gut-brain axis (5). In this respect, a recent review article by Nobis and colleagues (31) highlighted several biomarkers connected with neurotransmission, inflammation, and oxidative stress that are modulated in clinical depressive conditions such as major depressive disorder. While more work in needed to firmly establish the presence of such links in non-clinical populations, the need to further explore approaches that influence gut-brain axis activity and regulation are of distinct interest. In this regard, Edebol Carlman and colleagues (32) reported that 4 weeks of supplementing with a multi-strain probiotic was linked to increased functional connectivity between regions of the brain, with a seemingly greater response on the arithmetic stress test. However, this increased connectivity did not impact cortisol concentrations or measures of cognitive performance. Alternatively, Kazemi et al. (33) randomized 81 subjects with major depressive disorder to probiotic supplementation (10 × 10^9^ CFU *L. helveticus* R0052 and *B. longum* R01750) or placebo for 8 weeks and reported significant improvements in Beck Depression Inventory (BDI-II) scores and reductions in cortisol.

Serotonin, a neurotransmitter with hormone actions, is produced in the body and is closely associated with mood, stress, and anxiety (34). Approximately 95% of the body’s serotonin is produced in the gut (35). Connections between microbiome metabolites such as short-chain fatty acids and serotonin levels have been established, and as a result, probiotic administration has been used to modulate serotonin levels. For example, Riezzo and colleagues (36) randomized 56 patients with functional constipation to ingest Limosilactobacillus *reuteri* DSM 17938 or a placebo for 105 days to determine changes in serotonin and brain-derived neurotrophic factor. Serotonin levels decreased in response to supplementation while no changes were observed in the controls. Additional investigations examining *Lactiplantibacillus plantarum* DR7 have connected administration of this probiotic with improvements in serotonin pathway involvement (37). Other potential areas of efficacy for probiotics lies with their ability to support gut barrier function and integrity resulting in reductions in inflammation (38). This consideration is important as systemic inflammation is linked to the development and progression of many chronic health conditions. Systemic inflammation results from chronic activation of the immune system resulting in increased circulating levels of C-reactive protein, TNF-alpha, IL-6, and others (39–41). Ingestion of prebiotics, dietary fibers, and various probiotic strains (e.g., Bifido and lactobacillus) have been suggested to exert a favorable impact on short-chain fatty acid production and other inflammatory modulators through various mechanisms (19, 42, 43). In this respect, probiotics have previously been shown to prevent translocation of bacteria in the gut secondary to dysbiosis, which can help prevent immune cell activation and subsequent activation of inflammatory pathways (38). Romijn et al. (44) supplemented participants with *L. helveticus* R0052 (CNCM I-1722) and *B. longum* R0175 (CNCM I-3470) (≥3 × 10^9^ CFU/day) for 8 weeks and found that neither depression ratings or serum levels of key inflammatory cytokines (TNF-alpha, IL-6, IL-1B) were impacted. Alternatively, Akkasheh and colleagues (45) reported decreases in C-reactive protein and Beck Depression scores after supplementing for 8 weeks with *L. acidophilus*, *L. casei* and *B. bifidum* (6 × 10^9^ CFU/day).

While initial research in clinically stressed or depressed populations suggest probiotics may help to improve mood, depression, and anxiety, more research is needed to fully examine the potential of probiotic administration to impact mood, depression, personality, and anxiety including their ability to impact key hormones which may regulate mood, inflammation, and stress in healthy populations. As an individual’s mood, anxiety, personality, and stress levels can all intersect to influence how the brain and gut interact with each other, our study’s purpose was to examine the impact of supplementing with a multi-strain probiotic on changes in mood, depression, personality, and anxiety as well as circulating concentrations of cortisol, C-reactive protein, serotonin, and dopamine.

## Methods

### Overview of research design

This study followed a randomized, double-blind, placebo-controlled, parallel group design that was intended to evaluate the ability of a multi-strain probiotic to influence self-reported levels of mood, depression, anxiety, personality traits, and associated biomarkers (e.g., serotonin, cortisol, dopamine, and C-reactive protein). Healthy men and women between the ages of 18–50 years were assigned to one of two supplementation groups. The probiotic formulation (MSP) administered during the study was a blend of the four strains: *Lactiplantibacillus plantarum* LP01 (LMG P-21021), *Limosilactobacillus fermentum* LF16 (DSM 26956), *Lacticaseibacillus rhamnosus* LR06 (DSM 21981), and *Bifidobacterium longum* 04 (DSM 23233) blended with maltodextrin (2.5 g) all belonging to Probiotical S.p.A. collection. The clinical formula had a cell potency measured by Plate Count of >4 × 10^9^ Colony Forming Units (CFU)/dose. (Biolab Research Method 014-06). Cell potency of the samples was also measured by flow cytometer (ISO 19344:2015 IDF 232:2015) resulting in values of >4 × 10^9^ Active Fluorescent Units (AFU)/dose. The probiotic formulation was referred to 4 × 10^9^ live cells/dose. Placebo (PLA) was composed of pure maltodextrins (2.5 g). Prior to study engagement, all participants signed an IRB-approved informed consent document (Lindenwood University: IRB-19-212, approval date: 7/17/19, conformed to the standards set by the latest revision of the Declaration of Helsinki), and completed a health history questionnaire, and a series of additional questions to determine study eligibility.

Based upon previous data of Marotta et al. (30) *a priori* sample size evaluation indicated that a sample size of 28–33 participants would be needed. This estimation assumes an effect size of 0.5–0.55, with an alpha (α) level of 0.05 and estimated power (1 – β) of 0.80. Participants were scheduled for four identical study visits between 06:00 and 10:00 h while attempts were made to standardize visit timing across the investigation. For all visits, subjects were instructed to undergo an 8–10 h fast (except water) and abstain from strenuous physical activity 48 h prior to each visit. All participants supplemented for 6 weeks followed by a 3-week washout period post-supplementation. Participants donated a venous blood sample at 0 and 6 weeks of supplementation, and after a 3-week washout and were evaluated for changes in mood, personality, depression, and anxiety at: 0, 2, 4, and 6 weeks of supplement administration, and again post 3-week washout. Dietary records (2 days) were collected after 0, 2, 4, and 6 weeks of supplementation in addition to 3 weeks after ceasing supplementation. This study protocol and design was registered on Clinicaltrials.gov on April 25, 2022, as NCT05343533.^1^ Primary outcomes for this study were determined to be the mood-related questionnaires [e.g., Beck Depression Inventory (BDI-II), State/Trait Anxiety (STAI), Leiden Index of Depression Sensitivity-Revisited (LEIDS-R)] and biomarkers (e.g., cortisol, serotonin, dopamine, and C-reactive protein) while secondary outcomes were determined to the personality-related questionnaires [e.g., Cope Orientation to the Problems Experienced (COPE), Behavioral Inhibition System and Behavioral Activation System Scale (BIS/BAS), and the Life Orientation Test-Revised (LOT-R)] (Table 1).

**Table 1:** Overview of research design.

Week	Pre	0	2	4	6	9
Visit	1	2	3	4	5	6
Review and sign consent	X					
Answer study questions	X					
Food/fluid log		X	X	X	X	X
Take assigned supplement			X	X	X	
DEXA		X				
Psychological questionnaires		X	X	X	X	X
Adverse event monitoring		X	X	X	X	X
Biomarkers		X			X	X

### Study participants

**Figure 1:**
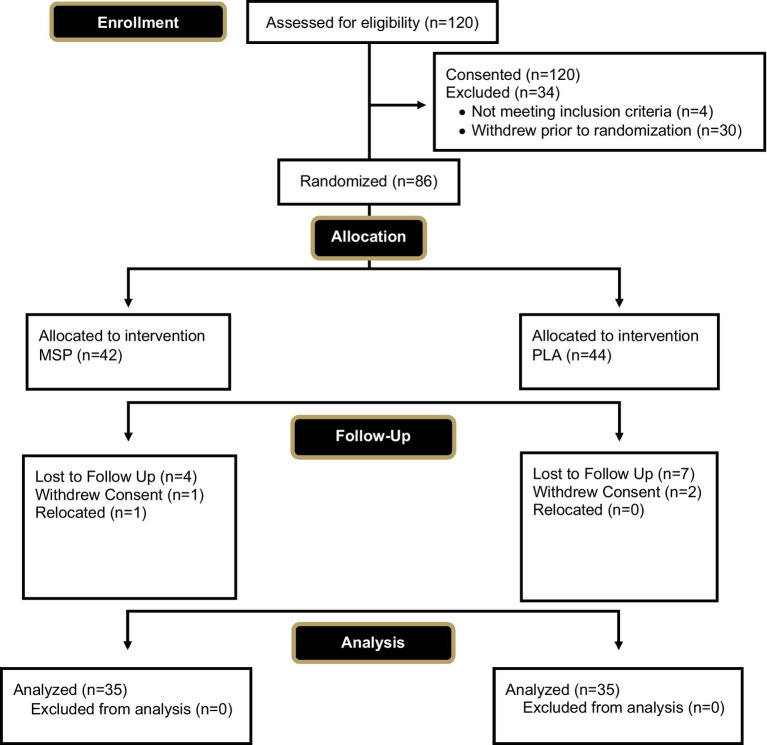
CONSORT diagram.

A total of 70 healthy men and women (31.0 ± 9.5 years, 173.0 ± 10.4 cm, 73.9 ± 13.8 kg, 26.7 ± 7.1% fat, 24.6 ± 3.5 kg/m^2^) successfully completed the study protocol. A Consolidation Standards of Reporting Trials (CONSORT) diagram is provided in Figure 1. Participants were included in the study if they were between the ages of 18–50 years, had a body mass index (BMI) between 18.5–32 kg/m^2^ (if BMI was 30–32 kg/m^2^, participant was required to have body fat percentage < 25% for men and < 35% for women for inclusion), were weight stable for the past 3 months (<5% variation in body mass), and deemed healthy through completion of a health history questionnaire. Participants were excluded if they were diagnosed or were being treated for any cardiac, respiratory, endocrine, psychiatric, musculoskeletal, renal, hepatic, neuromuscular or metabolic disease or disorder that precluded safe participation or would contraindicate quality control over the collected data, were diagnosed with or being treated for celiac disease, lactose intolerance, digestive insufficiencies, or other gastrointestinal complications such as irritable bowel syndrome, ulcerative colitis, etc., reported being a current smoker or had quit within the past 6 months, reported having used any illicit or recreational drugs including anabolic steroids within the past 30 days, reported the intake of any prescription or over-the-counter medications (i.e., antibiotics) that may impact study outcomes, reported the current use of any dietary supplements known to impact digestion or sleep quality for the past 30 days, reported taking a probiotic within the past 30 days, had been actively trying to lose weight, or were currently following a ketogenic or low carbohydrate diet within the past 30 days. Further, antibiotic use at any point in the study protocol led to removal from the study. Women who were pregnant, lactating, or indicated during screening they were actively trying to become pregnant were excluded from the study.

### Procedures

#### Baseline demographics and hemodynamics

Participants rested quietly for approximately 10 min before measuring resting heart rate and blood pressure (Omron BP785, Omron Corporation, Kyoto, Japan). Body mass was determined (Tanita BWB-627A, Tokyo, Japan) and recorded to the nearest ±0.1 kg. Height was measured using a standard wall-mounted stadiometer (Tanita, HR-200, Tokyo, Japan) and recorded to the nearest ±0.5 centimeter (cm).

#### Diet and physical activity controls

After the baseline visit and before each subsequent visit, study participants completed a hand-written two-day food record and were provided a copy to facilitate diet replication for the subsequent study period. In addition to the two-day food log, participants completed the automated self-administered 24-h dietary assessment tool (ASA24).^2^ Average energy, carbohydrate, fat, and protein intake were recorded and are presented in the current paper. In addition, Healthy Eating Index values were computed from the completed food records. The Healthy Eating Index is a measure of diet quality that can be used to assess compliance with the Dietary Guidelines for Americans. From the collected food intake information, 13 different food categories (seafood & plant proteins, fatty acids, refined grains, sodium added sugars, saturated fats, total fruits, whole fruits, total vegetables, greens & beans, whole grains, dairy, and total protein foods) are populated and the amount of food and fluid consumed that aligns with that food category is evaluated against the recommended intake.^3^ Each category is then assigned a percentage and from there a Healthy Eating Index composite score is determined. Radar plots have been generated to evaluate the difference in HEI values between groups and to evaluate the extent to which composite HEI values changed across time. Physical activity was not controlled throughout this study protocol. Eligible participants were instructed to maintain physical activity status throughout the study protocol, and to inform research staff if their physical activity habits changed throughout the study protocol.

#### Body composition (DEXA)

Body composition was obtained using dual-energy X-ray absorptiometry (DEXA). Calibration procedures were performed before each testing session and all scans were completed using a Hologic QDR Discovery A (HOLOGIC, Bedford, MA, USA) and analyzed using the accompanying software (Hologic APEX Software, Version 4.5.3; HOLOGIC) to obtain body composition parameters. The test–retest reliability (ICC and CV) of these procedures in 40 healthy college-aged men and women, was determined to be 0.99 and 1.26% for DEXA fat and 0.99 and 0.75% for DEXA fat-free mass (data not shown). All results were calculated using the NHANES correction factor.

#### Venous blood collection and processing

Blood samples were collected from the participants at weeks 0, and 6 weeks of supplementation, and after a three-week washout period. Each sample was collected using standard phlebotomy techniques into two serum separator (SST) and two ethylenediaminetetraacetic acid (EDTA) Vacutainer™ tubes. Following collection, tubes were gently inverted ten times. SST tubes were allowed to clot at room temperature for 30 min before being centrifuged, while EDTA tubes were immediately centrifuged at 4°C for 20 min at 2,000 revolutions per minute (rpm) (MegaFuge XFR, ThermoFisher Scientific, Waltham, MA, USA). After centrifugation, plasma and serum were aliquoted (600 μL) into separate micro-centrifuge tubes to minimize freeze–thaw cycles and labeled. Samples were then stored at -80°C for later analysis of serotonin, cortisol, dopamine, and C-reactive protein.

#### Mood-related questionnaires

##### Leiden Index of Depression Sensitivity – Revised

Leiden Index of Depression Sensitivity – Revised is a self-reported questionnaire that tests cognitive reactivity to sad mood (46). LEIDS-R consists of 34 statements describing different situations, and participants rated the extent to which each state applies to themselves.

##### State-Trait Anxiety Inventory

State-Trait Anxiety Inventory (STAI) is a self-reported questionnaire that measures the presence and severity of existing symptoms of anxiety and anxiety predisposition (47). STAI consists of two subscales, one for state anxiety (STAI – form Y1) and another for trait anxiety (STAI – form Y2). Each subscale consists of 20 questions. STAI – Y1 measures the current anxiety state by asking participants how they currently feel. STAI – Y2 measures anxiety predisposition by asking participants to rate how they generally feel in their life.

##### Beck Depression Inventory

Beck Depression Inventory is a self-reported questionnaire that measures the occurrence and severity of existing depressive symptoms (48). Participants chose statements that best describe their feelings over the past 2 weeks.

#### Personality-related questionnaires

##### Cope Orientation to the Problems Experienced

Cope Orientation to the Problems Experienced (COPE) is a self-reported questionnaire that evaluates coping strategies including cognitive and behavioral approaches people use to manage stressful situations (49). COPE includes 60 statements divided into five components: social support, avoidance strategies, positive attitude, problem-solving, and reliance on religion.

##### Behavioral Inhibition System and Behavioral Activation System Scale

Behavioral Inhibition System and Behavioral Activation System Scale is a self-reported questionnaire that assesses an individual’s sensitivity to behavioral inhibition and behavioral activation (50). The BIS/BAS consists of 24 statements related to punishment, reward anticipation, and control.

##### Life-Orientation Test-Revised

Life Orientation Test-Revised is a self-reported questionnaire that measures the dispositional optimism and pessimism (51) and consists of ten statements (*n* = 3 are worded positively, *n* = 3 are worded negatively, and *n* = 4 are control). Participants rated their agreement with statements regarding optimism and pessimism.

#### Biochemical analysis

Plasma samples were analyzed in duplicate using 96-well ELISA kits for serotonin, cortisol, dopamine, and C-reactive protein concentrations following the manufacturer’s instructions (DRG International, Springfield, NJ, USA). All samples exhibited absorbance values within the standard curve. Plate to plate controls were employed between all analyzed plates and exhibited coefficient of variations that ranged from 5 to 10% between the plates for each biomarker.

#### Supplementation protocol

Using a randomized, double-blind, placebo-controlled, parallel group fashion, participants were assigned to ingest a single capsule of either a maltodextrin PLA or a mixture of four probiotic strains (MSP). Each probiotic dose was delivered in capsules containing a 1 × 10^9^ live cells dose of each of the following strains (total daily dose of 4 × 10^9^ live cells): *Limosilactobacillus fermentum* LF16 (DSM 26956), *Lacticaseibacillus rhamnosus* LR06 (DSM21981), *Lactiplantibacillus plantarum* LP01 (LMG P-21021), and *Bifidobacterium longum* 04 (DSM 23233) (Probiotical S.p.A., Novara, Italy). Capsules for the placebo and probiotic strain were identical in color, shape, size, and transparency and were packaged into identical, bottled, containers that contained the same number of capsules. Participants were instructed to consume each dose at approximately the same time each day with 240–360 mL of water and within 2 h of consuming a meal. Product stability was monitored across the whole study up to 24 months at three different temperature conditions (refrigerated at 5°C, Zone II at 25°C and 65% relative humidity and Zone IVb at 30°C and 75% relative humidity) confirming the threshold values of 4 × 10^9^ CFU at the end of the 24 months program (See Supplementary Data).

#### Adverse event reporting

Adverse events were collected via spontaneous reporting by the participants, clinical evaluation, interaction of a research team member with a participant, or through review of a participant’s research file throughout the entire duration of the protocol. The frequency of each adverse event was recorded along with corresponding severity ratings of ‘mild’, ‘moderate’, or ‘severe’.

### Statistical analysis

Before any statistical tests were performed, data was screened for data entry and organization errors, and then analyzed for normality, skewness, and kurtosis. Any non-normally distributed value was normalized, if possible, using log-transformations. Parametric data is reported as means ± standard deviations while non-parametric data is reported as median ± interquartile range. For all statistical tests, data was considered statistically significant when the probability of type I error is 0.05 or less. A trend or a tendency for change was determined when the probability of type I error was *p* = 0.051 – ≤0.10. Parametric data was analyzed using mixed factorial ANOVA (group x time) with repeated measures on time. When significant group x time interactions were found, delta values (from baseline) were calculated and assessed using independent t-tests. Main effects of time were analyzed using single-factor ANOVA with repeated measures on time, and pairwise comparisons were evaluated using Bonferroni corrections. Non-parametric data was first assessed using a Friedman test for K-related samples, and if significant (*p* < 0.05), follow-up assessments were completed using a Wilcoxon signed-rank test between each baseline score and each subsequent follow-up timepoint. Bonferroni corrections were applied to evaluate pairwise comparisons (0.05 / # of comparisons being made). For all outcomes, *a priori* statistical approach was used to conduct a mixed gender analysis, as it was the primary aim identified for this investigation. When an outcome exhibited a significant group x time interaction, a three-way interaction was explored, including gender, to identify if any condition-specific outcomes were due to gender. Further, in a post-hoc fashion, changes in serotonin in participants assigned to MSP were dichotomized into ‘responders’ and ‘non-responders’, whereby responders were defined as those participants who exhibited an increase in circulating serotonin where non-responders exhibited no change or a decrease in circulating serotonin. Participants were then assigned a dummy code and the observed change in those outcomes where condition-specific differences were realized (Beck Depression, state anxiety, trait anxiety, LEDIS total, and LOT-R total score) and compared for differences. Pearson correlations were completed to evaluate the presence of any significant relationships. All analyses were completed using Microsoft Excel and the Statistical Package for the Social Sciences (v27; SPSS Inc.).

## Results

### Test product viability and stability

MSP was analyzed via plate count method as colony forming units (CFU) (Internal Method 014–06) and flow cytometry (ISO 19344, 2015: IDF 232: 2015) as Active Fluorescent Units (AFU) upon batch release (Biolab srl, Novara, Italy). To exclude product sample heterogeneity three random samples, withdrawn in triplicate during product manufacturing, were analyzed for total fluorescent units (TFU), and Relative Standard Deviation was <10%. Water activity of the product (aw) was monitored during the study to exclude any possible detrimental effects on probiotic cells or spoilage of the product due to the increase of water activity that was kept below 0.100. Product stability was monitored for 24 months at 5, 25°C and 30°C to be representative of a refrigerated condition, Zone II condition and Zone IVb conditions, respectively, according to ICH pharma guidelines.^4^ Figures of this data are provided as Supplementary Figures 1, 2.

### Supplementation compliance

Participants reported excellent compliance to each supplementation period. Compliance in the MSP condition ranged from 76.2 and 100.0% compliance (mean ± SD: 93.7 ± 7.3%) while compliance in the PLA group ranged from 71.4 and 100.0% compliance (mean ± SD: 94.6 ± 7.1%).

### Adverse event reporting

Adverse events were self-reported throughout the clinical trial. In totality, 12 mild or moderate adverse events were reported by participants assigned to the MSP condition (constipation, *n* = 1; tired in morning, *n* = 1, flatulence, *n* = 4, bloating, *n* = 3; lower back pain, *n* = 2; lower abdominal pain, *n* = 1) and 47 mild or moderate adverse events were reported by participants assigned to the PLA condition (constipation, *n* = 3; tired in morning, *n* = 1, burping, *n* = 3; flatulence, *n* = 6, bloating, *n* = 4; lower back pain, *n* = 2; lower abdominal pain, *n* = 1; acid reflux, *n* = 4; irritated esophagus, *n* = 2; skin irritation, *n* = 13, dry eyes, *n* = 4, fatigue, *n* = 2, and increased need to use bathroom, *n* = 1). No serious adverse events were reported.

### Dietary replication and hemodynamics

**Figure 2:**
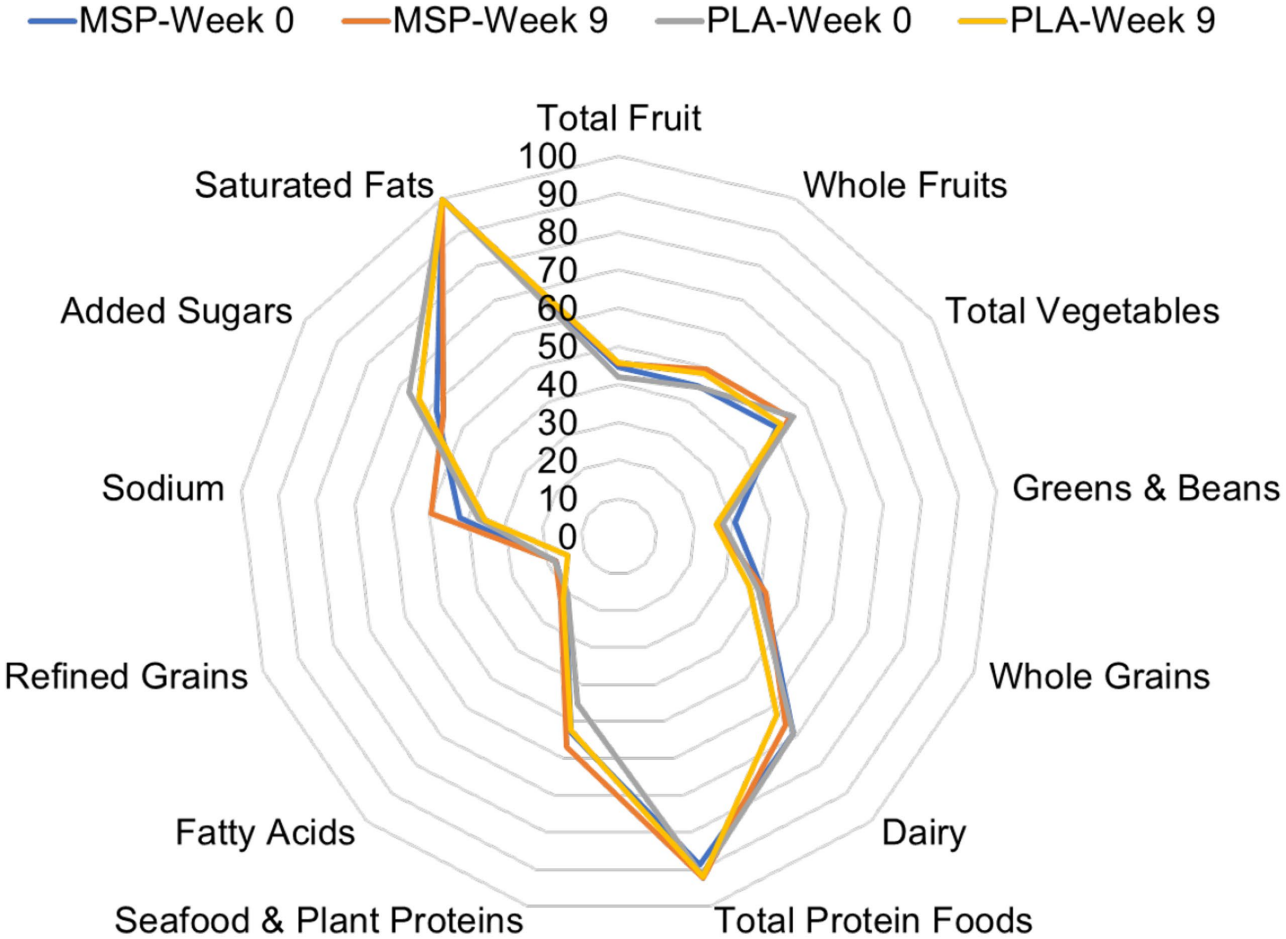
Radar plot of Healthy Eating Index values of MSP and PLA after 0 and 9 weeks of supplementation. MSP, Multi-strain probiotic; PLA, Placebo.

Of the 70 participants who completed the current study, 60 of them provided suitable dietary data. Four additional participants provided suitable baseline dietary data, but did not provide a suitable follow-up measure. For these four cases, missing data was replaced by carrying the baseline value forward. No differences were identified between groups for energy (MSP: 1891 ± 569 kcals/day vs. PLA: 2050 ± 618 kcals/day, *p* = 0.27), protein (MSP: 87.2 ± 31.8 grams/day vs. PLA: 97.2 ± 37.6 grams/day, *p* = 0.23), fat (MSP: 77.1 ± 26.2 grams/day vs. PLA: 88.5 ± 29.6 grams/day, *p* = 0.09), and carbohydrates (MSP: 212.3 ± 66.2 vs. PLA: 213.0 ± 76.7 grams/day, *p* = 0.97) throughout the study. Figure 2 is a radar plot that illustrates various Healthy Eating Index (HEI) components as well as the composite HEI score.

### Baseline characteristics

Table 2 provides age, height, body mass, percent body fat, and body mass index values at baseline for all participants (*n* = 70), men (*n* = 35), and women (*n* = 35). No differences between groups were present prior to beginning supplementation.

**Table 2:** Baseline characteristics.

		Group	Mean	(SD)	Minimum	Maximum	*p*
All subjects (*n* = 70)	Age	MSP	29.7	(9.0)	18	47	0.25
		PLA	32.3	(10.0)	18	50	
	Height (cm)	MSP	171.8	(10.6)	152	193	0.35
		PLA	174.2	(10.2)	148	195	
	Weight (kg)	MSP	71.9	(13.9)	50.6	107.5	0.23
		PLA	75.9	(13.6)	46.5	104.7	
	DXA % fat	MSP	26.5	(5.8)	13.2	37.3	0.88
		PLA	26.8	(8.3)	14.4	45.9	
	Body mass index (kg/m^2^)	MSP	24.3	(3.5)	18.8	32.5	0.41
		PLA	25.0	(3.5)	19.4	32.3	
Females (*n* = 35)	Age	MSP	28.0	(8.9)	18	47	0.03
		PLA	35.3	(10.2)	20	50	
	Height (cm)	MSP	165.7	(7.7)	152	181	0.50
		PLA	167.6	(8.5)	148	178.5	
	Weight (kg)	MSP	64.6	(9.7)	50.6	82.1	0.37
		PLA	67.8	(11.2)	46.5	88.9	
	DXA % fat	MSP	29.2	(4.9)	21.5	37.3	0.05
		PLA	33.0	(6.5)	22.5	45.9	
	Body mass index (kg/m^2^)	MSP	23.6	(3.8)	18.9	31.9	0.68
		PLA	24.1	(3.3)	19.4	31.8	
Males (*n* = 35)	Age	MSP	31.7	(9.0)	19	47	0.57
		PLA	29.8	(9.6)	18	48	
	Height (cm)	MSP	179.1	(9.0)	160	193	0.83
		PLA	179.7	(8.2)	156.5	195	
	Weight (kg)	MSP	80.6	(13.3)	59.5	107.5	0.62
		PLA	82.8	(11.8)	64	104.7	
	DXA % fat	MSP	23.4	(5.4)	13.2	34.1	0.32
		PLA	21.6	(5.5)	14.4	34.2	
	Body mass index (kg/m^2^)	MSP	25.1	(3.1)	20.5	31.4	0.60
		PLA	25.7	(3.5)	19.9	31.0	

Data is presented as mean (Standard Deviation); MSP, multi-strain probiotic; PLA, placebo; cm, centimetre; kg, kilogram; DXA, dual-energy x-ray absorptiometry; m^2^, meters squared.

### Questionnaires assessing mood-related constructs

**Figure 3:**
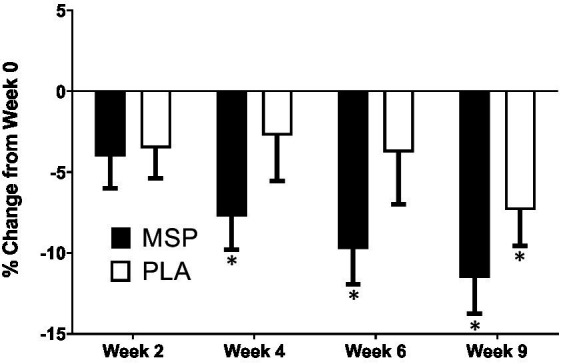
Percent change in Beck Depression Inventory scores. MSP, Multi-strain probiotic; PLA, placebo. *Indicates differences of specified time point from respective W0 (baseline) value using a Wilcoxon Signed-Rank Test (*p* < 0.05).

**Figure 4:**
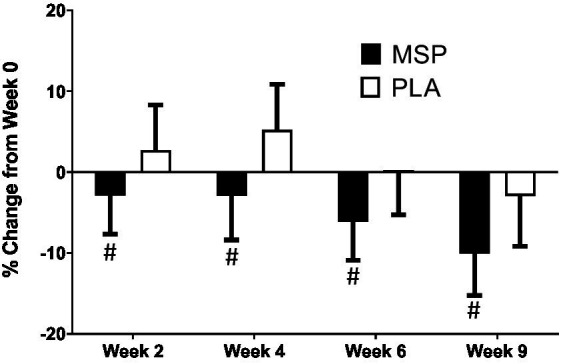
Percent change in Leiden Index of Depression Sensitivity (LEIDS) total score. MSP, Multi-strain probiotic; PLA, placebo. ^#^Indicates difference between groups using Mann–Whitney U at specified time point.

A significant time effect on Beck Depression Inventory (BDI-II) scores was observed in MSP (*p* < 0.001) as well as PLA (*p* = 0.007) using the Friedman test (Figure 3; Table 3). Follow-up analysis using Wilcoxon signed-rank tests in MSP indicated that reported scores for week 0 (W0) were different than scores for week 4 (W4, *p* = 0.001), week 6 (W6, *p* < 0.001), and 3 weeks after supplementation stopped (W9, *p* < 0.001). Follow-up analysis using Wilcoxon signed-rank tests in PLA indicated that reported scores for week 0 (W0) were different than scores for W9 (*p* < 0.001). No between-group differences (Mann–Whitney U) were observed at any of the five time points (*p* > 0.05). A significant three-way interaction (gender × group × condition) was observed (*p* = 0.035). Each gender was then analyzed separately. No difference was observed for BDI-II scores in females (Mean difference: 1.23 ± 1.69; 95% CI: −2.21, 4.68, *p* = 0.742) whereas a significant difference (−3.37 ± 1.31, 95% CI: −6.05, −0.70, *p* = 0.015) was identified in males. A significant time effect on state anxiety scores as measured in the State-Trait Anxiety Inventory (STAI) was observed in MSP (*p* < 0.001) and PLA (*p* = 0.04) using the Friedman test (Table 3). Follow-up analysis using Wilcoxon signed-rank tests with Bonferroni corrections applied for multiple comparison in MSP indicated that reported scores for week 0 (W0) were different than scores for W4 (*p* = 0.01), W6 (*p* < 0.001), and W9 (*p* = 0.001) and tended to be different for W2 (*p* = 0.02). Additionally, MSP scores were less than PLA (*p* = 0.04) after 6 weeks of supplementation. A significant time effect on trait anxiety scores was observed in MSP, (*p* < 0.001) while no difference was observed for PLA (*p* = 0.80) using the Friedman test (Table 3). Follow-up analysis using Wilcoxon signed-rank tests in MSP indicated that reported scores for week 0 (W0) were different than scores for W6 (*p* = 0.003) and tended to be different than W9 (*p* = 0.03). Results from the Mann–Whitney U test indicates that MSP scores were different than PLA at W0 (*p* = 0.02), W4 (*p* = 0.004), W6 (*p* = 0.005), and W9 (*p* = 0.003), while W2 scores tended (*p* = 0.06) to be different. No significant gender x group x condition was observed for state anxiety (*p* = 0.212) and trait anxiety (*p* = 0.117). Six subscales (hopeless/suicidality, acceptance/coping, aggression, control/perfectionism, risk aversion, and rumination) and a total score were assessed using the Leiden Index of Depress Sensitivity-Revised (LEIDS-R) (Figure 4; Table 3). No significant time effects were observed for the LEIDS-R hopeless/suicidality subscale for MSP (*p* = 0.10) and PLA (*p* = 0.10). No within-group differences were observed for either group using Wilcoxon signed-rank tests. Mann–Whitney U results indicated that MSP had significantly lower values than PLA at W0 (*p* = 0.02), W4 (*p* = 0.004), W6 (*p* = 0.006), and W9 (*p* = 0.003). To account for differences at baseline, each subsequent value was subtracted from its respective baseline value and using this approach no significant differences were found between conditions for the LEIDS-R hopeless subscale at W2 (= 0.811), W4 (*p* = 0.263), W6 (*p* = 0.306), and W9 (*p* = 0.725). No significant time effects were observed for the LEIDS-R acceptance/coping subscale for MSP (*p* = 0.07) and PLA (*p* = 0.62). To account for differences at baseline, each subsequent value was subtracted from its respective baseline value and using this approach no significant differences were found between conditions for the LEIDS-R acceptance subscale at W2 (= 0.952), W4 (*p* = 0.300), W6 (*p* = 0.194), and W9 (*p* = 0.168). No within-group differences were observed for either group using Wilcoxon signed-rank tests. No significant between group differences were observed as assessed by Mann–Whitney U tests. No significant time effects were observed for the LEIDS-R aggression subscale for MSP (*p* = 0.37) and PLA (*p* = 0.06). No within-group differences were observed for either group using Wilcoxon signed-rank tests. Mann–Whitney U results indicated that MSP had significantly lower values than PLA at W0 (*p* = 0.02), W2 (*p* = 0.04), W4 (*p* = 0.002), W6 (*p* = 0.03), and W9 (*p* = 0.02). To account for differences at baseline, each subsequent value was subtracted from its respective baseline value and using this approach no significant differences were found between conditions for the LEIDS-R aggression subscale at W2 (= 0.854), W4 (*p* = 0.080), W6 (*p* = 0.887), and W9 (*p* = 0.896). No significant time effects were observed for the LEIDS-R control/perfectionism subscale for MSP (*p* = 0.75) and PLA (*p* = 0.61). No within-group differences were observed (*p* > 0.05) for either group using Wilcoxon signed-rank tests. No between-group differences were observed (*p* > 0.05) using Mann–Whitney U at all time points. When Bonferroni corrections were applied for multiple comparisons, no significant time effects were observed for the LEIDS-R risk aversion subscale for MSP (*p* = 0.04) and PLA (*p* = 0.04). Wilcoxon signed-rank tests indicated that W9 values were different than W0 in MSP (*p* = 0.004) and PLA (*p* = 0.01). No between-group differences were observed (*p* > 0.05) using Mann–Whitney U at all time points. No significant time effects were observed for the LEIDS-R rumination subscale for MSP (*p* = 0.27) and PLA (*p* = 0.38). Wilcoxon signed-rank tests indicated that W6 values were different than W0 in MSP (*p* = 0.02), but no within-group differences were present in PLA (*p* > 0.05). Between-group differences were observed at W6 (*p* = 0.04) and W9 (*p* = 0.04) while values tended to be different between groups after W2 (*p* = 0.07) and W4 (*p* = 0.07). No significant time effects were observed for the LEIDS-R total score for MSP (*p* = 0.31) and PLA (*p* = 0.18). No within-group differences were observed (*p* > 0.05) for either group using Wilcoxon signed-rank tests. Between-group differences were observed at W0 (MSP: 36.3 ± 13.4 vs. PLA: 43.1 ± 14.2, *p* = 0.04), W2 (MSP: 34.5 ± 13.5 vs. PLA: 41.8 ± 13.4, *p* = 0.04), W4 (MSP: 35.1 ± 15.2 vs. PLA: 43.3 ± 15.1, *p* = 0.03), W6 (MSP: 33.5 ± 14.2 vs. PLA: 41.6 ± 15.9, *p* = 0.03) and W9 (MSP: 31.9 ± 13.6 vs. PLA: 39.7 ± 15.2, *p* = 0.03). To account for differences at baseline, each subsequent value was subtracted from its respective baseline value and using this approach no significant differences were found between conditions for the LEIDS-R total at W2 (= 0.796), W4 (*p* = 0.338), W6 (*p* = 0.424), and W9 (*p* = 0.981).

**Table 3:** Measured outcomes associated with mood-related constructs including the Beck Depression Inventory, State Anxiety, and Trait Anxiety, and the Leiden Index of Depression Sensitivity (LEIDS-R) after 0, 2, 4, and 6 weeks of supplementation as well as 3 weeks after stopping supplementation (week 9).

	Group	W0	W2	W4	W6	W9	Friedman (p)
Beck depression	MSP	24.67 (5.29)	23.40 (4.27)	22.49 (4.49)*	21.94 (4.20)*	21.37 (3.31)*	< 0.001
	PLA	24.54 (5.75)	23.43 (5.10)	23.43 (5.25)	23.09 (5.33)	22.29 (3.85)	0.007
Life orientation test-revisited	MSP	12.46 (1.79)	13.80 (2.34)*	13.40 (2.12)	13.20 (2.31)	13.60 (2.14)*	0.011
	PLA	12.80 (2.59)	13.03 (2.47)	13.37 (2.85)	12.77 (1.80)	13.31 (2.46)	0.666
BAS-control	MSP	5.43 (2.39)	4.78 (2.30)	4.94 (2.62)	4.66 (2.70)	4.34 (2.53)	0.14
	PLA	5.41 (2.88)	5.40 (2.52)	5.22 (2.52)	4.71 (2.42)	4.94 (2.52)	0.58
BAS-drive	MSP	8.57 (2.49)	8.29 (2.38)	8.11 (2.37)	8.43 (2.40)	7.74 (2.52)	0.03
	PLA	8.22 (2.49)	8.11 (2.41)	7.37 (2.00)*	7.60 (2.45)	7.94 (2.79)	0.01
BAS-fun seeking	MSP	8.06 (1.89)	7.80 (2.03)	7.71 (2.18)	7.74 (2.19)	7.31 (2.27)*	0.16
	PLA	7.69 (2.22)	7.66 (2.17)	7.23 (1.93)	7.34 (2.25)	7.54 (2.34)	0.11
BAS-reward responsiveness	MSP	8.00 (1.66)	7.83 (1.77)	8.09 (1.98)	8.06 (2.22)	7.49 (2.11)	0.25
	PLA	7.34 (1.80)	7.43 (1.88)	7.37 (1.90)	7.54 (1.99)	7.51 (2.15)	0.71
BIS	MSP	14.26 (2.43)	14.63 (2.49)	14.09 (2.23)	14.54 (2.16)	14.29 (2.58)	0.36
	PLA	15.09 (2.36)	14.91 (2.72)	14.57 (2.84)	15.00 (3.04)	15.69 (3.05)	0.13
State anxiety	MSP	32.5 (9.1)	30.6 (8.3)	30.0 (9.5)*	28.1 (8.0)*#	28.4 (9.2)*	< 0.001
	PLA	33.2 (9.4)	33.3 (9.5)	30.9 (9.2)	32.1 (9.7)	31.6 (9.2)	0.04
Trait anxiety	MSP	34.6 (9.8)#	34.6 (10.2)	33.4 (9.8)#	32.2 (8.8)*#	33.0 (9.8)#	< 0.001
	PLA	37.1 (9.4)	36.9 (9.0)	36.7 (9.2)	36.6 (9.5)	35.7 (8.5)	0.80
LEIDR-S: hopelessness/suicidality	MSP	3.31 (2.82)#	2.91 (2.93)	2.86 (2.99)#	2.40 (2.26)*#	2.40 (2.28)*#	0.10
	PLA	4.60 (2.87)	3.97 (3.16)	4.66 (3.16)	4.40 (3.09)	4.11 (2.54)	0.10
LEIDS-R: acceptance/coping	MSP	2.77 (2.18)	3.06 (2.67)	3.63 (3.41)	3.91 (3.33)	3.40 (3.23)	0.07
	PLA	2.91 (2.77)	3.11 (2.55)	3.23 (2.68)	3.29 (3.13)	2.97 (2.78)	0.62
LEIDS-R: aggression	MSP	4.46 (4.10)#	3.82 (3.01)#	4.00 (3.02)#	4.14 (3.21)#	3.89 (3.15)#	0.37
	PLA	6.60 (4.37)	6.17 (4.91)	7.31 (4.93)	6.51 (4.77)	6.54 (5.03)	0.06
LEIDS-R: control/perfectionism	MSP	8.34 (3.86)	8.09 (3.65)	8.37 (4.14)	7.91 (4.43)	7.57 (4.04)	0.75
	PLA	9.14 (3.66)	8.97 (3.73)	9.14 (4.03)	9.11 (3.94)	8.49 (4.16)	0.61
LEIDS-R: risk aversion	MSP	9.17 (3.37)	8.94 (3.71)	8.69 (3.54)	8.11 (3.33)	7.60 (3.53)*	0.04
	PLA	10.49 (3.47)	9.97 (2.56)	9.91 (3.10)	9.29 (3.56)	8.71 (3.17)*	0.04
LEIDS-R: sensitivity/rumination	MSP	8.26 (3.60)	7.69 (3.24)	7.51 (3.65)	7.03 (3.80)#	7.03 (4.09)#	0.27
	PLA	9.34 (4.24)	9.57 (4.33)	9.06 (3.83)	9.03 (3.86)	8.83 (4.07)	0.38
LEIDS-R: total score	MSP	36.3 (13.4)	34.5 (13.5)	35.1 (15.2)	33.5 (14.2)	31.9 (13.6)	0.31
	PLA	43.1 (14.2)	41.8 (13.4)	43.3 (15.1)	41.6 (15.9)	39.7 (15.2)	

MSP, multi-strain probiotic; PLA, placebo; W0, Week 0; W2, Week 2; W4, Week 4; W6, Week 6; W9, Week 9; Data is presented as median (interquartile range); * Indicates differences of specified time point from respective W0 (baseline) value using a Wilcoxon Signed-Rank Test (*p* < 0.05); # Indicates difference between groups using Mann–Whitney U at specified time point (*p* < 0.05).

### Questionnaires assessing personality-related constructs

A significant time effect on LOT-R scores was observed in MSP (*p* = 0.011), but not PLA (*p* = 0.066) using the Friedman test (Table 4). Follow-up analysis using Wilcoxon signed-rank tests in MSP indicated that reported scores for week 0 (W0) were different than scores for week 2 (W2, *p* = 0.003) and W9 (*p* = 0.001) and were different from W4 (*p* = 0.03). No between-group differences (Mann–Whitney U) were observed at any of the five time points (*p* > 0.05). No significant gender x group x condition was observed for LOT-R total scores (*p* = 0.128). Four subscales of the behavioral activation scale (control, drive, fun seeking, reward responsiveness) (BAS) and the behavioral inhibition scale (BIS) were evaluated (Table 4). A significant time effect for BAS-Drive scores was observed in MSP (*p* = 0.03) and PLA (*p* = 0.01) using the Friedman test (Table 4). Follow-up analysis using Wilcoxon signed-rank tests in PLA indicated that reported scores for W4 (*p* = 0.003) were different than W2, while no other within-group differences were reported in this condition. No within-group differences between time points were observed in MSP. No significant time effect was observed for MSP or PLA for the control, fun seeking, and rewards responsiveness subscales or BIS. Further, no between-group differences (Mann–Whitney U) were observed at any of the five time points (*p* > 0.05) for any of the four BAS subscales and for the BIS. A significant time effect was observed for MSP scores for Cope Orientation to the Problems Experienced (COPE) mental disengagement subscale (*p* = 0.005). For PLA, significant time effects were observed for the COPE behavioral disengagement (*p* = 0.02), restraint (*p* = 0.01), use of emotional social support (*p* = 0.003), and acceptance (*p* = 0.04) subscales. Within-group changes in MSP were observed between W0 and W4 (*p* = 0.02), W6 (*p* = 0.004), and W9 (*p* = 0.003) for the COPE mental disengagement subscale. Within-group changes in PLA were observed between W0 and W4 for the behavioral disengagement (*p* = 0.009), use of emotional social support (*p* = 0.03) and restraint (*p* = 0.007) subscales, between W0 and W6 for the restraint (*p* = 0.01) and acceptance (*p* = 0.01) subscales, and between W0 and W9 for the restraint subscale (*p* = 0.02). Between-group differences were observed for the COPE behavioral disengagement subscale at W4 (*p* = 0.03), W6 (*p* = 0.05), and W9 (*p* = 0.04).

**Table 4:** Measured outcomes associated with mood-related constructs Life Orientation Test-Revisited (LOTR), Behavioral Activation (BAS), Behavioural Inhibition (BIS), and the Cope Orientation to the Problems Experienced (COPE) after 0, 2, 4, and 6 weeks of supplementation as well as 3 weeks after stopping supplementation (week 9).

Construct	Group	W0	W2	W4	W6	W9	Friedman (p)
LOTR	MSP	12.46 (1.79)	13.80 (2.34)*	13.40 (2.12)	13.20 (2.31)	13.60 (2.14)*	0.011
	PLA	12.80 (2.59)	13.03 (2.47)	13.37 (2.85)	12.77 (1.80)	13.31 (2.46)	0.666
BAS-control	MSP	5.43 (2.39)	4.78 (2.30)	4.94 (2.62)	4.66 (2.70)	4.34 (2.53)	0.14
	PLA	5.41 (2.88)	5.40 (2.52)	5.22 (2.52)	4.71 (2.42)	4.94 (2.52)	0.58
BAS-drive	MSP	8.57 (2.49)	8.29 (2.38)	8.11 (2.37)	8.43 (2.40)	7.74 (2.52)	0.03
	PLA	8.22 (2.49)	8.11 (2.41)	7.37 (2.00)*	7.60 (2.45)	7.94 (2.79)	0.01
BAS-fun seeking	MSP	8.06 (1.89)	7.80 (2.03)	7.71 (2.18)	7.74 (2.19)	7.31 (2.27)*	0.16
	PLA	7.69 (2.22)	7.66 (2.17)	7.23 (1.93)	7.34 (2.25)	7.54 (2.34)	0.11
BAS-reward responsiveness	MSP	8.00 (1.66)	7.83 (1.77)	8.09 (1.98)	8.06 (2.22)	7.49 (2.11)	0.25
	PLA	7.34 (1.80)	7.43 (1.88)	7.37 (1.90)	7.54 (1.99)	7.51 (2.15)	0.71
BIS	MSP	14.26 (2.43)	14.63 (2.49)	14.09 (2.23)	14.54 (2.16)	14.29 (2.58)	0.36
	PLA	15.09 (2.36)	14.91 (2.72)	14.57 (2.84)	15.00 (3.04)	15.69 (3.05)	0.13
COPE-positive reinterpretation and growth	MSP	12.29 (2.26)	12.57 (2.38)	12.31 (2.30)	12.20 (2.65)	12.23 (2.73)	0.93
	PLA	12.60 (2.42)	12.83 (2.23)	12.80 (2.17)	12.86 (2.25)	12.91 (2.49)	0.46
COPE-mental disengagement	MSP	8.97 (2.35)	9.11 (2.48)	8.31 (2.56)*#	8.11 (2.30)*#	7.83 (1.98)*#	0.005
	PLA	8.69 (1.75)	9.00 (1.96)	9.11 (1.97)	9.06 (1.97)	9.11 (1.84)	0.55
COPE-focus on and venting of emotions	MSP	8.62 (3.24)	8.54 (3.12)	8.89 (3.22)	8.29 (2.83)	8.23 (2.86)	0.29
	PLA	8.00 (2.83)	8.89 (2.72)	9.31 (3.41)	9.29 (3.14)	9.17 (3.25)	0.18
COPE-use of instrumental social support	MSP	11.23 (2.78)	10.91 (2.55)	10.97 (3.14)	11.03 (2.96)	11.09 (3.17)	0.79
	PLA	11.54 (2.44)	11.17 (2.86)	12.03 (2.57)	11.71 (2.79)	11.54 (2.95)	0.20
COPE-active coping	MSP	11.89 (2.18)	11.83 (2.12)	11.69 (2.72)	11.83 (2.44)	11.86 (2.53)	0.56
	PLA	12.14 (2.68)	11.89 (2.19)	12.09 (2.28)	12.29 (2.31)	11.94 (2.34)	0.61
COPE-denial	MSP	11.89 (2.18)	11.83 (2.12)	11.69 (2.72)	11.83 (2.44)	11.86 (2.53)	0.37
	PLA	12.14 (2.68)	11.89 (2.19)	12.09 (2.28)	12.29 (2.31)	11.94 (2.34)	0.43
COPE religious coping	MSP	8.91 (4.88)	9.03 (4.93)	8.86 (5.13)	9.00 (5.11)	9.00 (5.26)	0.98
	PLA	9.74 (4.40)	9.86 (4.36)	10.03 (4.50)	9.74 (4.46)	9.97 (4.53)	0.51
COPE-humor	MSP	10.26 (3.13)	10.63 (3.12)	10.03 (3.76)	9.91 (3.31)	9.71 (3.61)	0.27
	PLA	9.74 (2.66)	9.51 (3.39)	9.94 (3.24)	9.91 (3.50)	9.74 (3.76)	0.67
COPE-behavioral disengagement	MSP	5.57 (1.70)	5.71 (2.02)	5.54 (1.62)	5.14 (1.68)	5.17 (1.60)	0.08
	PLA	5.69 (1.53)	6.00 (1.39)	6.43 (1.80)*#	5.63 (1.42)#	5.94 (1.80)#	0.02
COPE-restraint	MSP	9.83 (2.42)	9.77 (2.72)	9.86 (2.69)	10.03 (2.50)	9.69 (2.05)	0.75
	PLA	9.00 (2.06)	9.51 (1.93)	9.77 (2.16)*	9.74 (2.20)*	9.74 (2.11)*	0.01
COPE-use of emotional social support	MSP	10.37 (3.54)	10.20 (3.20)	10.94 (3.57)	11.06 (3.31)	10.91 (3.36)	0.14
	PLA	10.37 (3.54)	10.20 (3.20)	10.94 (3.57)*	11.06 (3.31)	10.91 (3.36)	0.003
COPE-substance use	MSP	4.51 (1.48)	4.57 (1.77)	4.46 (1.22)	4.31 (0.83)	4.49 (1.12)	0.83
	PLA	4.83 (2.04)	4.74 (1.65)	4.80 (1.57)	4.66 (1.57)	4.77 (1.78)	0.73
COPE-acceptance	MSP	11.86 (2.29)	11.97 (2.77)	11.43 (2.80)	11.29 (2.92)	10.89 (3.44)	0.15
	PLA	11.23 (2.21)	11.63 (2.40)	11.91 (2.32)	12.06 (2.47)*	11.60 (2.42)	0.04
COPE-suppression of competing activities	MSP	9.31 (2.14)	9.77 (2.13)	9.34 (2.44)	9.43 (2.33)	9.11 (2.25)	0.15
	PLA	9.29 (2.05)	9.23 (2.06)	9.94 (1.75)	9.83 (2.02)	9.43 (1.75)	0.08
COPE-planning	MSP	12.46 (2.58)	12.51 (2.33)	12.29 (2.37)	12.31 (3.20)	12.06 (2.65)	0.91
	PLA	12.34 (2.78)	12.60 (2.48)	12.60 (2.50)	12.83 (2.43)	12.54 (2.68)	0.86

MSP, multi-strain probiotic; PLA, placebo; W0, Week 0; W2, Week 2; W4, Week 4; W6, Week 6; W9, Week 9; Data is presented as median (interquartile range); * Indicates differences of specified time point from respective W0 (baseline) value using a Wilcoxon Signed-Rank Test (*p* < 0.05); # Indicates difference between groups using Mann–Whitney U at specified time point (*p* < 0.05).

### Biomarkers assessment

No significant group x time interactions were observed for serotonin (*p* = 0.77), cortisol (*p* = 0.48), C-reactive protein (*p* = 0.45), and dopamine (*p* = 0.23). A significant time effect was observed for serotonin (*p* = 0.03) while no significant main effects for time were observed for dopamine (*p* = 0.71) and cortisol (*p* = 0.89), although observed changes in C-reactive protein tended to change (*p* = 0.052) across the study protocol. Simple main effects analysis indicated that serotonin levels significantly increased in MSP after W6 (*p* = 0.03) and W9 (*p* = 0.02) while no changes in serotonin were observed in PLA. Independent samples t-tests were completed to evaluate if serotonin responders and non-responders exhibited different changes in reported scores for BDI-II, state anxiety, trait anxiety, LEIDS-R total score, and LOT-R total. In all situations, no significant differences were found between the changes in these questionnaires and serotonin (*p* = 0.301–0.540). No significant correlations were found between the changes in serotonin and the observed changes in BDI-II Inventory (*r* = 0.143, *p* = 0.412), state anxiety (*r* = 0.078, *p* = 0.656), trait anxiety (*r* = 0.099, *p* = 0.572), LEIDS-R total score (*r* = −0.041, *p* = 0.817), and LOT-R total score (*r* = −0.101, *p* = 0.566).

## Discussion

The aim of the present study was to evaluate the efficacy of multi-strain probiotic supplementation on circulating biomarkers and associated changes in mood, depression, and anxiety in healthy adults. Our results indicate that probiotic supplementation significantly improved outcomes related to depression, anxiety, and mood, alongside increases in serotonin concentrations, however, the probiotic supplementation had limited or no ability to impact outcomes related to associated coping mechanisms during stressful situations and plasma concentrations of cortisol, C-reactive protein, and dopamine. Previous work in clinical and non-clinical populations have provided evidence that probiotic supplementation can favorably impact mood, depression, and anxiety (29, 52, 53), however these outcomes are not universal (54–57). In particular, Marotta et al. (30) implemented a similar study design and used the same probiotic multi-strain formula as the present study and, reported improvements in the profile of mood states (POMS) questionnaire as well as LEIDS-R scale for acceptance. Results from the present study expands upon these findings whereby scores from three out of the six LEIDS-R subscales (hopeless, aggression, rumination), and the total score were significantly lower than scores observed for PLA (Figure 4). The positive changes in cognitive reactivity after MSP supplementation in the present study are also supported by the previous findings of Steenbergen et al. who supplemented 40 healthy adults with a multi-species probiotic for 4 weeks. In contrast to Marotta et al. (30) and Steenbergen et al. (29), the present investigation demonstrated significant improvements in depression scores as evaluated by the BDI-II (Figure 4). Unlike the studies of Marotta et al. (30) and Steenbergen et al. (29) was, the average values for the BDI-II could qualitatively be interpreted as our participants being mildly depressed (Table 3). In addition to the changes reported in mood and depression, changes in state and trait anxiety from supplementation were also evaluated. Participants supplementing with MSP reported significant reductions in state anxiety when compared to baseline, while no changes were reported across the protocol for PLA. Similarly, trait anxiety scores in MSP were lowered at W6 and tended to remain at W9. When compared to values reported in PLA, the MSP group reported significantly lower trait anxiety scores (Table 3). Of special note, the clinical trial was initiated in early 2020 prior to the outbreak of the COVID-19 global pandemic and was subsequently restarted in September 2020. The extent to which the stress and anxiety associated with the pandemic was impacted by our study design remains to be fully determined, but nonetheless our results were realized when many countries and societies were grappling with the challenges and struggles associated with the pandemic.

Changes in various questionnaires that focused on personality-associated outcomes were examined in the present study with several of these measures suggesting that MSP lacks application for altering these outcomes. For example, of the 15 subscales in the COPE questionnaire (Coping Orientation to Problem Experienced), no changes were identified for ten of the subscales (Table 4). For those subscales that were altered, the observed changes were within only one group, apart from Behavioral Disengagement, whereby PLA indicated they were more likely to use this coping mechanism than MSP at W4, W6, and W9. Similarly, no between-group differences were realized for the Behavioral Activation (BAS) and Inhibition (BIS) scales while limited changes across time were identified for the Drive and Fun Seeking subscales (Table 4). Overall, these results align with the previous work of Marotta et al. (30) where limited changes in the COPE and no changes in BIS/BAS were also reported. Finally, and somewhat in contrast to Marotta et al. (30), the MSP group reported significant changes in LOT-R scores which suggested that participants who supplemented with MSP adopted a more optimistic orientation as the protocol progressed. No such changes were identified in PLA and no differences between groups were found for LOT-R scores.

In response to supplementation, changes in concentrations of serotonin, dopamine, cortisol, and C-reactive protein in the blood were evaluated. No changes were identified for cortisol, C-reactive protein, or dopamine; however, serotonin concentrations were significantly increased in MSP (Table 5), while no changes were observed in PLA. These changes are valuable as they provide a mechanistic link to the changes observed in mood, depression, and anxiety questionnaires, albeit the exact mechanism remains to be further determined. Serotonin is a key neurotransmitter linked to various psychological and psychiatric states (34), while more than 95% of the body’s serotonin is produced in the gut (35). However, one must consider that plasma serotonin is not able to cross the blood–brain barrier and the reader should not interpret our findings to suggest the increased serotonin can directly interact with the brain. Instead, we posit as others have (30) that the administered probiotic regimen altered the intestinal microbiota composition in a manner that impacted the production of neurotransmitter precursors, short-chain fatty acids, or other secondary metabolites (23, 24) that went on to impact the regulation or production of various substances that modulated brain functions resulting in our observed changes in the mood-related questionnaires in the present study. Importantly, our findings support previous investigations demonstrating the ability of a MSP to improve mood and cognitive reactivity (29, 30), while extending these findings to demonstrate that changes may be associated with increases in serotonin due to supplementation. Previous work by Karbownik and colleagues (58), contrasts the findings from the present study, where a decrease in salivary serotonin in 32 healthy medical students supplementing with either a placebo or *Saccharomyces boulardii* CNCM I-1079 for 30 days prior to a stressful event was reported. However, a higher cohort age, a longer supplementation regimen, and the administration of multiple strains versus one strain in the present investigation, should be considered when comparing these outcomes. With regards to cortisol, several investigations using similar study designs, have similarly reported limited potential for probiotics to impact cortisol levels (54, 59).

**Table 5:** Serotonin, dopamine, cortisol, and C-reactive protein after 0 and 6 weeks of supplementation as well as 3 weeks after stopping supplementation (week 9).

	Group	W0	W6	W9		*p*
Serotonin	MSP	73 (49)	111 (114)*	116 (117)*	Group	0.38
	PLA	99 (76)	131 (132)	122 (129)	Time	0.03
					Group × Time	0.77
Dopamine	MSP	55.2 (25.8)	51.4 (26.9)	52.3 (24.8)	Group	0.88
	PLA	50.4 (22.3)	51.8 (29.6)	54.0 (27.8)	Time	0.71
					Group × Time	0.23
Cortisol	MSP	202 (99)	200 (90)	212 (101)	Group	0.008
	PLA	160 (58)	161 (57)	156 (58)	Time	0.89
					Group × Time	0.48
C-Reactive Protein	MSP	1.39 (1.47)	1.53 (1.73)	1.91 (2.00)	Group	0.73
	PLA	1.22 (1.49)	1.63 (2.05)	1.57 (2.14)	Time	0.052
					Group × Time	0.45

MSP, multi-strain probiotic; PLA, placebo; W0, Week 0; W6, Week 6; W9, Week 9; Data is presented as mean (Standard Deviation); * Indicates differences of specified time point from respective W0 (baseline) repeated measures ANOVA (*p* < 0.05).

A specific strength for our protocol was that it utilized a randomized, double-blind, placebo-controlled approach, with a mixed gender cohort. As seen with Marotta et al. (30), the 6-week supplementation and 3-week washout period allows for additional insight into the impact of the probiotic regimen, including a brief time period after supplementation ceased. Future research should explore the potential impact of longer supplementation and washout periods. Additionally, the combination of applied outcomes from several psychobiological domains in conjunction with systemic biomarkers to evaluate potential relationships between applied outcomes mechanisms should be considered an investigational strength. Some limitations of our study should be acknowledged. First, our protocol was not adequately powered to properly evaluate any potential gender differences, as the *a priori* analysis was completed with the intent of examining the efficacy of the combination of probiotic strains however, the mixed gender cohort increased external validity. Nevertheless, Beck depression scores between men and women were significantly different. Future investigations should explore this relationship more thoroughly using an adequately powered design. Additionally, a more extensive investigation into the dimensions of cognitive processing and personality could help elucidate areas in which probiotics may exert the greatest impact. We did not control for any dietary considerations in this study protocol except for monitoring compliance to the supplementation regimen. From the food records collected, we were able to determine that each group did not significantly change the amount of energy or macronutrients consumed throughout the study protocol. We also calculated Healthy Eating Index values from our food records and further determined that the amount of various food groups consumed by each group did not change across the study protocol. While it is possible that certain specific foods were consumed that contained prebiotics and synbiotics prior to beginning the study and throughout the study, this seems unlikely considering our evaluation of our dietary data. One of the primary underlying mechanisms to explain the changes we observed in the mood-related questionnaires was the notion that our probiotic supplementation regimen altered the composition of the intestinal microbiota which impacted the production of neurotransmitter precursors that were involved in mood disposition, etc. We did not collect fecal samples throughout our investigation and as a result this suggestion is only based upon speculation from previous findings (5, 23, 24). As such, these samples would have been an effective tool to evaluate changes in the gut microbiome and to potentially demonstrate the supplementation protocol’s ability to alter the presence and richness of candidate bacterial communities. Future investigations should consider examining fecal samples, in addition to systemic blood biomarkers, and applied psychological investigational tools to further explore the potential widespread impact of probiotic supplementation.

## Conclusion

In conclusion, the present study determined that in a cohort of 70 healthy men and women, a six-week regimen of a MSP consisting of four probiotic strains: *Limosilactobacillus fermentum* LF16 (DSM 26956), *Lacticaseibacillus rhamnosus* LR06 (DSM 21981), *Lactiplantibacillus plantarum* LP01 (LMG P-21021), and *Bifidobacterium longum* 04 (DSM 23233) providing a total dosage of 4 × 10^9^ live cells/day favorably impacted reported scores on validated questionnaires assessing depression, anxiety, and mood, while increasing plasma concentrations of serotonin.

## Data availability statement

The raw data supporting the conclusions of this article will be made available by the authors, without undue reservation.

## Ethics statement

The studies involving humans were approved by Lindenwood University, Protocol IRB-19-212, approval date: July 17, 2019. The studies were conducted in accordance with the local legislation and institutional requirements. The participants provided their written informed consent to participate in this study.

## Author contributions

CK, RJ, and MP: conception or design of the study. KW, AH, JK, JM, CG, LA, PM, and CK: acquisition of data and data collection. CK, PM, and AH: analysis and interpretation of data. All authors: drafting work and providing critical edits. All authors approved final version and agree to be held accountable for its contents.

## Funding

This study was co-sponsored by Probiotical S.p.A. (Italy) and Ashland (USA). The sponsors had no role in collecting the data, analyzing the data, interpreting the results, or preparing the manuscript.

## Conflict of interest

MP works for Probiotical srl. MP played no part in conducting, analyzing, interpreting, or preparing the manuscript for publication. RJ is a scientific advisor to Ashland, a co-sponsor with this study and works for Increnovo LLC. He assisted with study design, but played no part in collecting, analyzing, or interpreting the results of this study.

The remaining authors declare that the research was conducted in the absence of any commercial or financial relationships that could be construed as a potential conflict of interest.

## Publisher’s note

All claims expressed in this article are solely those of the authors and do not necessarily represent those of their affiliated organizations, or those of the publisher, the editors and the reviewers. Any product that may be evaluated in this article, or claim that may be made by its manufacturer, is not guaranteed or endorsed by the publisher.

## Footnotes

^1^ https://clinicaltrials.gov/ct2/show/NCT05343533

^2^ https://epi.grants.cancer.gov/asa24/

^3^ https://epi.grants.cancer.gov/hei/interpret-visualize-hei-scores.html

^4^ https://www.ema.europa.eu/en/ich-q1a-r2-stability-testing-new-drug-substances-drug-products-scientific-guideline
