# Corrigendum: A randomized controlled trial to examine the impact of a multi-strain probiotic on self-reported indicators of depression, anxiety, mood, and associated biomarkers

**DOI:** 10.3389/fnut.2023.1324536

**Published:** 2023-11-21

**Authors:** Kylie E. Walden, Jessica M. Moon, Anthony M. Hagele, Leah E. Allen, Connor J. Gaige, Joesi M. Krieger, Ralf Jäger, Petey W. Mumford, Marco Pane, Chad M. Kerksick

**Affiliations:** ^1^Exercise and Performance Nutrition Laboratory, Department of Kinesiology, College of Science, Technology, and Health, Lindenwood University, Saint Charles, MO, United States; ^2^Increnovo LLC, Milwaukee, WI, United States; ^3^Probiotical srl, Novara, Italy

**Keywords:** probiotic, gut-brain, quality of life, mood, serotonin

In the published article, there was an error. The viability results of the provided probiotic did not include reference to the live cell viability assessments that were also completed.

A correction has been made to the Abstract. This sentence previously stated:

“In a randomized, double-blind, placebo-controlled fashion, 70 healthy men and women (31.0 ± 9.5 years, 173.0 ± 10.4 cm, 73.9 ± 13.8 kg, 24.6 ± 3.5 kg/m^2^) supplemented with a single capsule of MSP (a total daily dose of 4 x 10^9^ colony forming units [CFU] comprised of a 1 x 10^9^ CFU dose from each of the following strains: *Limosilactobacillus fermentum* LF16, *Lacticaseibacillus rhamnosus* LR06, *Lactiplantibacillus plantarum* LP01, and *Bifidobacterium longum* 04, Probiotical S.p.A., Novara, Italy) or a maltodextrin placebo (PLA).”

The corrected sentence appears below:

“In a randomized, double-blind, placebo-controlled fashion, 70 healthy men and women (31.0 ± 9.5 years, 173.0 ± 10.4 cm, 73.9 ± 13.8 kg, 24.6 ± 3.5 kg/m^2^) supplemented with a single capsule of MSP (a total daily dose of 4 × 10^9^ live cells comprised of a 1 × 10^9^ live cells dose from each of the following strains: *Limosilactobacillus fermentum* LF16, *Lacticaseibacillus rhamnosus* LR06, *Lactiplantibacillus plantarum* LP01, and *Bifidobacterium longum* 04, Probiotical S.p.A., Novara, Italy) or a maltodextrin placebo (PLA).”

In the published article, there was an error. The viability results of the provided probiotic did not include reference to the live cell viability assessments that were also completed. We also failed to specify composition of the placebo.

A correction has been made to the Abstract. This sentence previously stated:

“They either received a multi-strain probiotic (1 x 10^9^ CFU of each of the four strains: *Limosilactobacillus fermentum* LF16 (DSM 26956), *Lacticaseibacillus rhamnosus* LR06 (DSM 21981), *Lactiplantibacillus plantarum* LP01 (LMG P-21021), and *Bifidobacterium longum* 04 (DSM 23233); Probiotical S.p.A., Novara, Italy) or a placebo (PLA).”

The corrected sentence appears below:

“The probiotic formulation (MSP) administered during the study was a blend of the four strains: *Lactiplantibacillus plantarum* LP01 (LMG P-21021), *Limosilactobacillus fermentum* LF16 (DSM 26956), *Lacticaseibacillus rhamnosus* LR06 (DSM 21981), and *Bifidobacterium longum* 04 (DSM 23233) blended with maltodextrin (2.5 g) all belonging to Probiotical S.P.A. collection. The clinical formula had a cell potency measured by Plate Count of >4 × 10^9^ Colony Forming Units (CFU)/dose. (Biolab Research Method 014-06). Cell potency of the samples was also measured by flow cytometer (ISO 19344:2015 IDF 232:2015) resulting in values of >4 × 10^9^ Active Fluorescent Units (AFU)/dose. The probiotic formulation was referred to 4 × 10^9^ live cells/dose. Placebo (PLA) was composed of pure maltodextrins (2.5 g).”

In the published article, there was an error. The viability results of the provided probiotic did not include reference to the live cell viability assessments that were also completed.

A correction has been made to the Abstract. This sentence previously stated:

“Each probiotic dose was delivered in capsules containing a 1 × 10^9^ colony forming units (CFU) dose of each of the following strains (total daily dose of 4 × 109 CFU): *Limosilactobacillus fermentum* LF16 (DSM 26956), *Lacticaseibacillus rhamnosus* LR06 (DSM21981), *Lactiplantibacillus plantarum* LP01 (LMG P-21021), and *Bifidobacterium longum 04* (DSM 23233) (Probiotical S.p.A., Novara, Italy).”

The corrected sentence appears below:

“Each probiotic dose was delivered in capsules containing a 1 × 10^9^ live cells dose of each of the following strains (total daily dose of 4 × 10^9^ live cells): *Limosilactobacillus fermentum* LF16 (DSM 26956), *Lacticaseibacillus rhamnosus* LR06 (DSM21981), *Lactiplantibacillus plantarum* LP01 (LMG P-21021), and *Bifidobacterium longum* 04 (DSM 23233) (Probiotical S.p.A., Novara, Italy).”

In the published article, there was an error. The viability results of the provided probiotic did not include reference to the live cell viability assessments that were also completed.

A correction has been made to the Abstract. This sentence previously stated:

“The finished supplementation product was analyzed (Biolab srl, Novara, Italy) via flow cytometry (ISO 19344, 2015: IDF 232: 2015) upon batch release which resulted in a cell count of >4 × 109 Active Fluorescent Unit (AFU)/g and plate count method as colony forming units (CFU) (Internal Method 014–06)”

The corrected sentence appears below:

“MSP was analyzed via plate count method as colony forming units (CFU) (Internal Method 014–06) and flow cytometry (ISO 19344, 2015: IDF 232: 2015) as Active Fluorescent Units (AFU) upon batch release (Biolab srl, Novara, Italy).”

The authors apologize for these errors and state that this does not change the scientific conclusions of the article in any way. The original article has been updated.

