# Supplementary figures and images for: A randomized controlled trial to examine the impact of a multi-strain probiotic on self-reported indicators of depression, anxiety, mood, and associated biomarkers
Source: Front Nutr. 2023 Aug 31;10:1219313. doi: 10.3389/fnut.2023.1219313 (PMC10501394; doi:10.3389/fnut.2023.1219313)

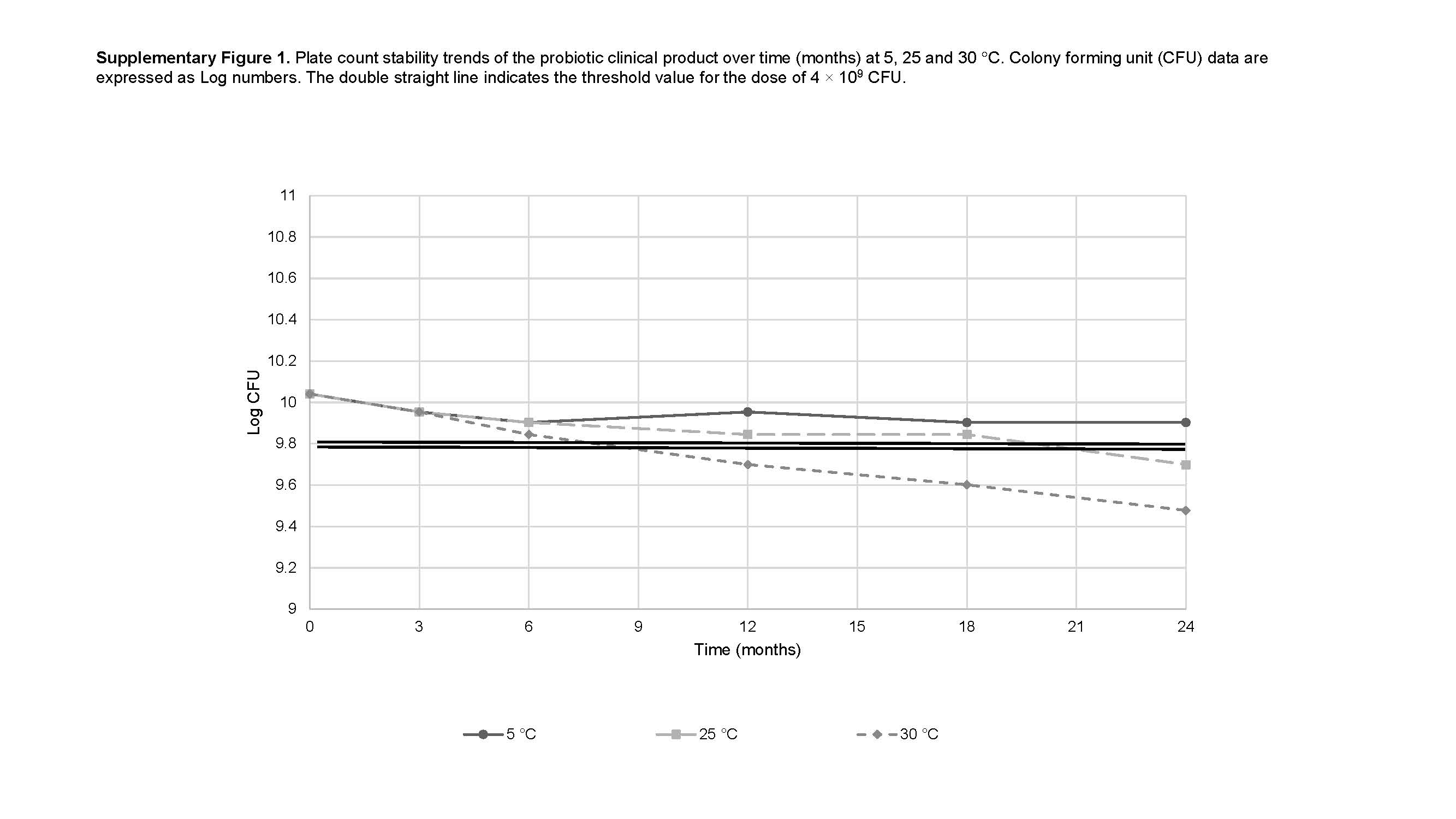

Supplement: Supplementary file 1 [file Image_1.jpg]

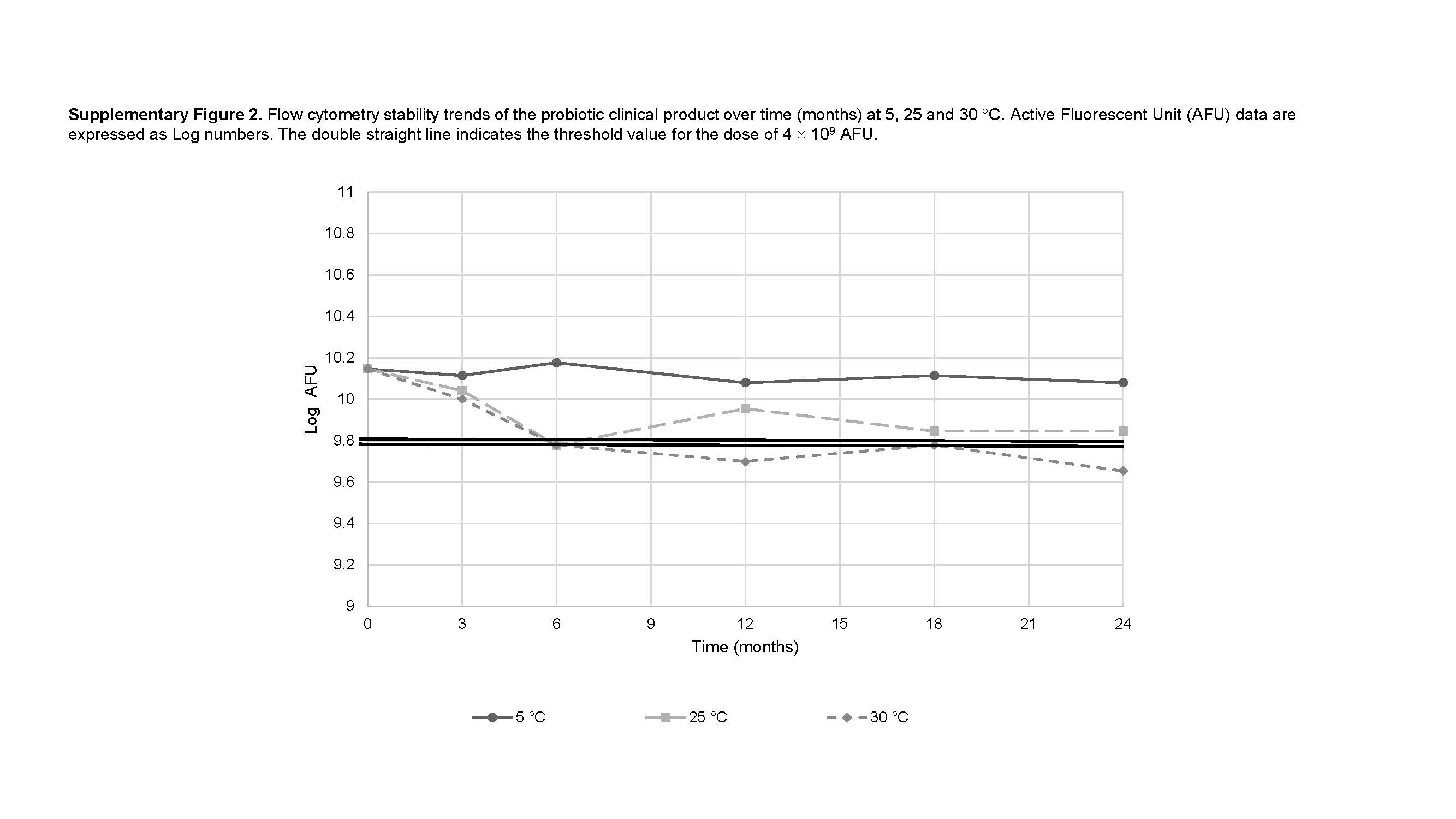

Supplement: Supplementary file 2 [file Image_2.jpg]
